# Addressing BBB Heterogeneity: A New Paradigm for Drug Delivery to Brain Tumors

**DOI:** 10.3390/pharmaceutics12121205

**Published:** 2020-12-11

**Authors:** Jessica I. Griffith, Sneha Rathi, Wenqiu Zhang, Wenjuan Zhang, Lester R. Drewes, Jann N. Sarkaria, William F. Elmquist

**Affiliations:** 1 Department of Pharmaceutics, University of Minnesota, Minneapolis, MN 55455, USA; rathi029@umn.edu (S.R.); zhan4937@umn.edu (W.Z.); zhan6717@umn.edu (W.Z.); 2 Department of Biomedical Sciences, University of Minnesota Medical School—Duluth, Duluth, MN 55812, USA; ldrewes@d.umn.edu; 3 Department of Radiation Oncology, Mayo Clinic, Rochester, MN 55902, USA; sarkaria.jann@mayo.edu

**Keywords:** blood-brain barrier, BTB, NVU/BBB, brain metastases

## Abstract

Effective treatments for brain tumors remain one of the most urgent and unmet needs in modern oncology. This is due not only to the presence of the neurovascular unit/blood–brain barrier (NVU/BBB) but also to the heterogeneity of barrier alteration in the case of brain tumors, which results in what is referred to as the blood–tumor barrier (BTB). Herein, we discuss this heterogeneity, how it contributes to the failure of novel pharmaceutical treatment strategies, and why a “whole brain” approach to the treatment of brain tumors might be beneficial. We discuss various methods by which these obstacles might be overcome and assess how these strategies are progressing in the clinic. We believe that by approaching brain tumor treatment from this perspective, a new paradigm for drug delivery to brain tumors might be established.

## 1. Introduction

The blood–brain barrier (BBB) remains one of the greatest obstacles to effective pharmaceutical interventions in the treatment of central nervous system (CNS) disease, including brain tumors. While it is true that some loss of neurovascular and barrier integrity may occur in and around brain tumors, the magnitude of this change is not consistent, and new pharmaceutical strategies for the treatment of brain tumors have yet to show significant efficacy in the clinic [1,2,3]. This lack of efficacy is largely attributed to insufficient drug delivery due to the presence of the BBB. The dense vascular network of the brain works to strictly regulate the transport of substances into and out of the brain parenchyma in order to maintain ionic homeostasis, nutrient supply, and removal of waste for optimal neuronal function. In recent decades, research has revealed that the BBB is composed of specialized endothelial cells (ECs), which are surrounded and supported by pericytes and astrocytes and are regulated by neuronal signaling, forming what is referred to as the neurovascular unit (NVU) [4]. A lack of vesicular transport across these specialized ECs and the presence of active efflux proteins help to further restrict the access of drugs to the CNS [5]. Currently, treatment for the majority of brain tumors involves maximal surgical resection, if possible, followed by radiation, and in the case of glioblastoma multiforme (GBM), concomitant temozolomide (TMZ) [3]. However, these treatments often prove to be palliative, and malignant brain tumors are nearly always fatal within five years of initial diagnosis [6,7].

While treatments for peripheral malignancies have improved dramatically in recent decades with the advent of earlier diagnosis, improved imaging, targeted small molecule inhibitors, and large molecule biologics, the treatment of brain tumors has lagged far behind, and their incidence is on the rise [7]. Therefore, it is imperative to understand how the NVU/BBB may be altered in the case of brain tumors and how to design pharmaceutical interventions specifically to overcome this challenge while maintaining neurovascular integrity as much as possible. To this end, a number of strategies have been proposed to improve drug delivery to the brain and brain tumors. Invasive strategies to bypass the NVU/BBB include convection-enhanced delivery (CED) and direct injection, in addition to polymer-based, biodegradable implants for drug delivery. Noninvasive strategies might include focused ultrasound (FUS) and hyperosmotic disruption of the NVU/BBB, as well as inhibition of efflux transporters, nanoparticle-based strategies, and the use of endogenous transport mechanisms across the brain EC by receptor-mediated transcytosis. In this review, we will introduce brain barrier anatomy and physiology, discuss the heterogeneous impacts of tumor growth and signaling on NVU/BBB integrity, and provide brief overviews of the strategies investigated to deliver drugs to CNS tumors.

## 2. Barriers and Boundaries in the Brain

Rational drug delivery to any organ requires a thorough understanding of the structures and properties of the target tissue. This section includes detailed features of the dynamic NVU model that is rapidly supplanting the former static BBB concept. Furthermore, the role that these features serve in guiding the molecular basis for current therapies for CNS tumors is illustrated.

### 2.1. CNS Blood–Tissue Barriers

Any strategy for blood-borne drug delivery into the CNS must consider several structural obstacles related to blood–tissue interfaces [8,9]. First, the brain is covered by layers of cells collectively described as the dura–arachnoid–pia membranes. The dura separates peripheral vessels within the cranium from the cerebrospinal fluid (CSF) in which the brain resides. Several compact layers of epithelial cells, with tight junctional contacts and relatively low surface areas, prevent materials from traversing this boundary. Within the CSF compartment, the pial layer contains vessels that penetrate the brain parenchyma. These vessels also exhibit very low permeability and surface area. Other major barriers include relatively small regions of CNS circulation and include the choroid plexus, circumventricular organs (CVOs), and ependymal cells. Each of these has specialized epithelial cells with properties that highly restrict water-soluble chemicals from penetrating the mass of parenchymal tissue. The choroid plexus that produces CSF, for example, contains specialized epithelial cells with tight junctions (TJs) encasing a fenestrated vascular endothelium that allows the exchange of blood constituents with the extracellular space. The CVOs are also composed of fenestrated endothelial cells, but their location is confined by surrounding tanycytes, a specialized epithelial cell that also has tight junctional contacts that localize and restrict the interstitial fluid. Direct exchange between the CSF and interstitial fluid is restricted by ependymal cells that line the ventricular surface and form a selectively permeable cellular barrier. 

### 2.2. Neurovascular Unit

**Figure 1:**
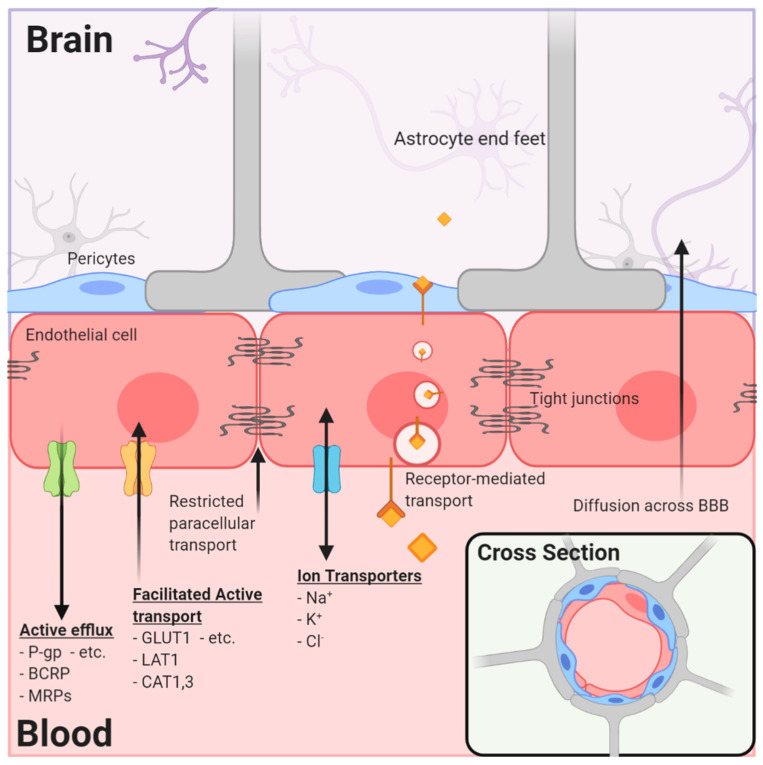
The neurovascular unit/blood–brain barrier (NVU/BBB) is composed of specialized endothelial cells and support cells, including pericytes and astrocytes. The cross-sectional view illustrates that the majority of the abluminal surface of the endothelial cell is covered by pericytes and astrocytic foot processes. Paracellular transport across the BBB/NVU is restricted by tight junction proteins, and even small, lipophilic molecules that might diffuse across the BBB may be subject to active efflux by a variety of proteins. Facilitated active transport, receptor-mediated transport, and ion transporters allow the brain to be supplied with nutrients while maintaining strict homeostasis.

By far, the major blood–tissue interface in the brain is the microvasculature network extending from arterioles to capillaries and to venules. By illustration, in one gram of human brain, the length of vessels, if joined end to end, approximates the length of 4.5 football fields and almost a quarter (~23%) of the surface area of a sheet of photocopy paper. The current model of the brain microvasculature consists of several different cell types working collectively to form a functional NVU [4,10]. The endothelial cells and their tight junctional contacts that block paracellular diffusion are surrounded by pericytes that form gap junctions with multiple adjacent endothelial cells and by astrocytic endfeet that cover >99% of the endothelial–pericyte cell surface (Figure 1). The astrocytes, in turn, extend processes that monitor synaptic activity and react by signaling endothelial cells and pericytes to respond to increased metabolic demands by increasing nutrient delivery. Microglia, the resident immune cells, are extravascular when dormant but react swiftly to remove cellular debris (phagocytosis) or respond to inflammatory signals associated with disease or injury. Loss of pericytes and their signaling in the NVU by injury or genetic means leads to reduced expression of endothelial tight junction proteins and dysfunction of their permeability barrier [11,12]. These examples illustrate the remarkable dynamics, plasticity, and interdependence of signaling among the NVU cells to maintain functional stability in the contemporary model of the neurovasculature.

### 2.3. Blood to Brain Permeability and Transport

As the brain depends on external nutrients for growth and development and yet is protected from the influence of circulating toxins or xenobiotics, the endothelial cell exhibits critical membrane-imbedded proteins that function as transporters. One group of transporters includes facilitated carriers and secondary active transporters for the delivery of energy substrates and essential nutrients (Table 1). The glucose transporter (GLUT1), monocarboxylic acid transporter (MCT1), and amino acid transporters are examples of the >40 transporters detected by functional and transcriptomic analyses [13]. 

A second type of transporter that is critical to brain drug delivery is the ABC (ATP-binding cassette) superfamily that uses the energy of ATP hydrolysis to expel endogenous and exogenous xenobiotics from the cell (and from the brain) and return them to the blood for transformation and excretion (Table 2). The most relevant ABC transporters expressed by brain endothelial cells are P-glycoprotein (P-gp, ABCB1), breast-cancer-related protein (BCRP, ABCG2), and the multiple drug-related proteins (MRP1, -4, -5; ABCC1, -4, -5). Many anticancer drugs are substrates of the ABC transporters and, therefore, may influence their effectiveness as brain cancer drugs (Figure 1). The relevance of ABC transporters in anticancer drug delivery in brain tumors with apparently high permeability is illustrated by the fact that the most permeable tumor vasculatures have influx rate constants several-fold less than the rate constants for other organs such as muscle, heart, lung, kidney, and liver [14]. Therefore, efflux mechanisms are likely important even in CNS tumors, in which the permeability of the brain vasculature is compromised and elevated compared to the surrounding tissue.

## 3. Heterogeneous Blood–Tumor Barrier Permeability

**Figure 2:**
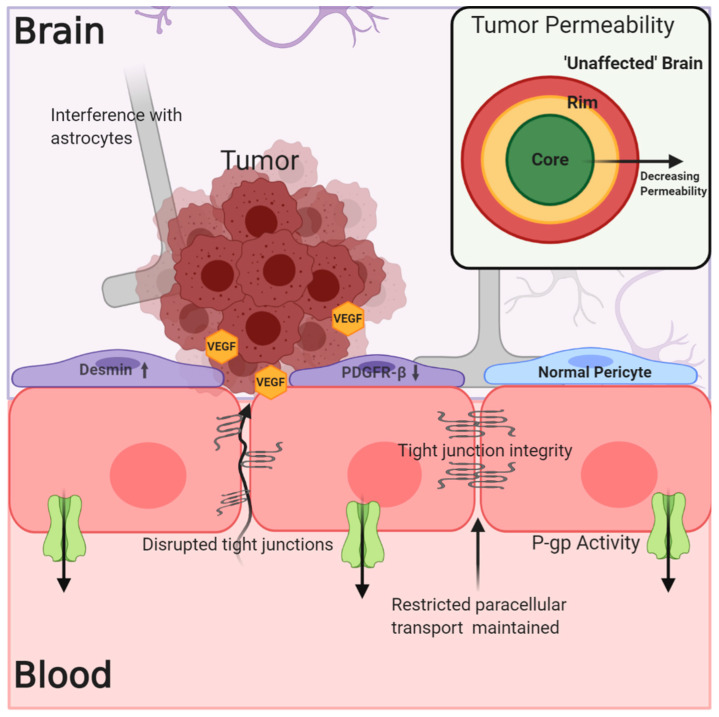
The blood–tumor barrier (BTB) is characterized by increased cytokine and VEGF signaling from the tumor, which may lead to decreased expression of tight junction (TJ) proteins like claudin-5. Alterations in pericyte phenotype and disruption of astrocytic associations with endothelial cells may contribute to decreased barrier integrity. However, this is not a uniform phenomenon within or among tumors, and the expression of efflux transporters limits drug permeation into the tumor. Evidence exists showing decreased permeability of the BTB in regions distant to the core of the tumor, which more closely resemble “unaffected” brain.

The understanding of the BBB’s physical and biochemical barrier functions, including the expression of tight junction proteins, restricted paracellular transport, and active efflux mechanisms, has been well established. However, determining the integrity of the NVU/BBB in and around tumors and how this affects tumor treatment has been less straightforward. In the case of both primary and metastatic tumors, the NVU/BBB is subject to changes due to tumor growth and signaling, and these alterations in NVU/BBB integrity and physiology result in what will hereafter be referred to as the blood–tumor barrier (BTB). The BTB may be characterized by an inflammatory environment with increased numbers of activated astrocytes, vascular endothelial growth factor (VEGF)-induced reduction in the expression of tight junction proteins like claudin-5, breakdown of the basal lamina, and tumor cell interference in associations between endothelial cells and astrocytic endfeet (Figure 2) [15,16,17]. There is also evidence for a change in the phenotype of BTB-associated pericytes, which may show decreased platelet-derived growth factor receptor-β (PDGFR-β) expression in addition to increased desmin expression [18]. As a result of these changes, the BTB can be, on average, somewhat “leakier” (more permeable) than the normal NVU/BBB in the absence of disease [17,19,20]. The predominant question with regards to BBB breakdown and the treatment of brain tumors has therefore been, is the breakdown of the NVU/BBB in the case of brain tumors significant and uniform enough to allow for the accumulation of efficacious drug concentrations?

As this question has been repeatedly investigated, various preclinical tumor models have routinely led to conflicting results. In some cases, tumor vascular permeability, assessed by the accumulation of fluorescent tracers, has been previously correlated with growth patterns, tumor size, or peripheral tumor of origin [21]. In other cases, including a variety of brain-trophic metastatic breast cancer models developed at the National Institutes of Health (NIH), no correlation between tumor size and permeability has been found [14,19]. These studies also found that the variability of BTB permeability among tumors in the same animal and even among regions of the same tumors, as assessed by the accumulation of fluorescent tracers and small molecules like paclitaxel, doxorubicin, and lapatinib, could be as much as 100-fold [14,22,23]. More recent studies in HER2+ brain-trophic breast cancer metastasis models have shown a poor correlation between drug accumulation and tracer accumulation, as well as inconsistent drug uptake and variable efficacy of biologics like trastuzumab and other antibody-based therapies [22,24,25]. Another model of lung cancer brain metastases found two-fold increases in permeability to small molecules like 3H-mannitol but concluded that this small relative increase in addition to functional P-gp was still a significant limitation to systemic drug therapy [26]. In addition, a number of studies utilizing transporter-knockout mice and patient-derived xenograft (PDX) models of GBMs and brain metastases have shown that the efficacy of systemic administration of various small molecules is consistently limited by the presence of the NVU/BBB and BTB, active efflux, and the fact that vascular permeability is widely variable within and around the tumor region [20,27,28,29,30,31,32,33]. This heterogeneity in permeability at the BBB leads to wide variability in drug/tracer accumulation and has also been confirmed by elegant correlated ultramicroscopy and MRI techniques in preclinical tumor models [34]. These studies point to the conclusion that relying on the potential for increased BTB permeability is unlikely to result in efficacious treatment through the systemic administration of novel therapies and their subsequent regulatory approval for such applications.

Although the aforementioned evidence has been largely preclinical, it agrees with clinical observations when considered in the appropriate context. Increased permeability of the BTB, relative to normal brain, is observed clinically, as increased uptake of tracers in magnetic resonance imaging (MRI) and positron emission tomography (PET) imaging allows for definitive diagnosis of brain tumors and informs many aspects of their treatment [35]. However, especially in the case of diffuse and invasive tumors like GBM, it has also been shown that nonenhancing, infiltrating regions of brain tumors often exist outside of the region of T1-weighted contrast enhancement [36,37]. This indicates that some portions of the malignant tumor are protected by a relatively uncompromised NVU/BBB. The patterns of treatment failure are strongly correlated with and attributed to these nonenhancing regions, and maximal resection that includes these regions improves survival [38,39,40]. Increasingly, early-phase studies, in which patients receive drugs prior to tumor resection and biopsy, are being utilized to determine the real extent of antineoplastic drug permeability to the BTB [1,2]. Although fold-increases in drug concentrations relative to normal brain may be observed at the core of the tumor, this may still not be adequate to cause cell death. As has been evidenced in many of the aforementioned preclinical models, it is unlikely that these drug concentrations are representative of concentrations in the entirety of the tumor. In the case of GBM, the infiltrative boundaries of the tumor are likely to have a more competent and intact BTB, closer to that of “unaffected brain” [20,31,41,42]. This heterogeneous drug distribution among different regions of the tumor is also clinically evidenced in drug concentrations from biopsies of non-contrast-enhancing tumor regions [43]. 

As there has been a great success with novel treatments of peripheral disease, the culmination of decades of brain tumor research has led to the conclusion that it is imperative that molecules and delivery strategies be designed foremost with an intact NVU/BBB in mind. As an example, GNE317, a small molecule that was designed specifically to avoid active efflux, showed significantly higher activity in a model of brain metastases of lung cancer than another counterpart PI3K inhibitor not designed to penetrate the BBB/NVU [44,45]. Other brain-penetrant inhibitors like osimertinib, an EGFR inhibitor, have also shown better preclinical and potential clinical efficacy [46,47]. While designing small lipophilic molecules in an attempt to optimize tumor penetration and minimize active efflux is certainly one potential method towards effective treatments for brain tumors, there are a vast number of other drug delivery strategies and novel molecules in development for this application. These strategies will be discussed in the following sections.

## 4. Invasive Technologies

**Figure 3:**
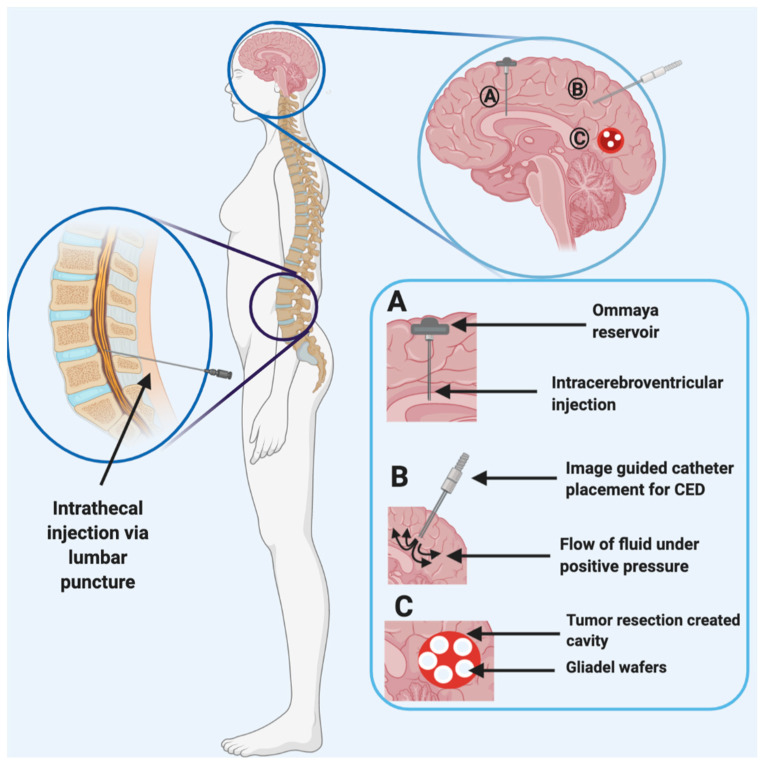
Various invasive technologies to increase drug delivery to the brain by bypassing the BBB/NVU include intrathecal injection via a lumbar puncture and various intracranial techniques. These include (**A**) intracerebroventricular injection using the Ommaya reservoir, (**B**) convection-enhanced delivery (CED) by way of intracerebral catheter placement, and (**C**) placement of drug-loaded polymeric wafers.

The NVU/BBB poses numerous challenges for efficient drug delivery to the brain and brain tumors, as discussed in the previous section. To address these challenges, various invasive and noninvasive strategies have been developed to improve the delivery of therapeutic agents to the brain. Invasive technologies are based on local delivery of therapeutics to the brain, bypassing the NVU/BBB entirely. They include drug delivery to the cerebrospinal fluid (CSF) via intrathecal or intraventricular injections and interstitial delivery via biodegradable wafers or catheters (Figure 3).

### 4.1. Intrathecal and Intraventricular Injections

Intrathecal (IT) administration involves direct injection of therapeutics into the CSF that fills the thecal space and encompasses intrathecal–lumbar injection but can also be used to describe intracerebroventricular or intracisternal magna injections [48]. Chemotherapy may be administered directly into the lumbar thecal sac via lumbar puncture or infused into the lateral ventricle through a subcutaneous reservoir and a ventricular catheter, allowing the drug to distribute into the target sites via diffusion [49]. Drug delivery via lumbar puncture may require multiple administrations, is highly invasive, causes discomfort to the patient, and is not likely to allow effective drug delivery to brain tumors. Alternatively, intraventricular infusions are often administered via the Ommaya reservoir, invented in 1963 by Ayub Ommaya, which is inserted into one of the lateral ventricles (Figure 3) [50,51]. Clinically, it is essential to ensure correct placement of the catheter in the ventricle, and, to this end, new state of the art technology using smartphones is being developed as a guide for accurate neuronavigation and catheter placement, which may make these procedures more accessible to a variety of clinics [52]. 

In the landscape of brain tumor treatment utilizing this technique, there are several clinical reports of intrathecal (IT) administration of large molecules, including trastuzumab, in patients with leptomeningeal metastatic brain tumors [53,54,55,56,57,58,59]. However, these studies also utilized concomitant systemic administration of trastuzumab, and, therefore, the positive symptomatic benefit cannot be solely attributed to IT administration. There are other case reports that have combined trastuzumab with other cytotoxic agents like methotrexate and/or cytarabine, resulting in prolongation of disease control in HER2+ brain tumor patients [60,61,62,63,64,65,66]. In the preclinical setting, prophylactic IT administration of adeno-associated virus serotype 9 (AAV9) gene therapy vectors and tumor tropic neural stem cells (NSCs) loaded with chemotherapeutic agents shows that IT administration has the potential for efficient drug delivery of large molecules and biological vectors to the subarachnoid space [48,67,68,69]. 

From amongst small molecules, methotrexate and cytarabine are frequently prescribed for IT administration. However, there are numerous reports of neurotoxicity and other complications such as transverse myelopathy associated with the IT administration of these drugs [70,71,72,73,74,75]. Therefore, although high concentrations can be attained in the CSF using IT injections, reducing the total dose and risk of systemic toxicity, this method of administration has its drawbacks. The rate of drug distribution is slow and inversely proportional to the molecular weight, meaning large molecules often have very low or undetectable concentrations when distant from the site of injection [76]. Additionally, rapid CSF turnover, as compared to the rate of diffusion, results in faster clearance of the therapeutics from the site of administration [77,78]. For years, there has been a common misconception that the distribution of drugs into the CSF is indicative of NVU/BBB permeability and that delivery of drugs to the CSF would ensure delivery to the deeper brain tissues. However, it is now more widely accepted that this is not the case, and the reader is directed to a review of this topic [79]. 

### 4.2. Convection-Enhanced Delivery

Convection-enhanced delivery (CED) is one of the most explored techniques to bypass the NVU/BBB; it was developed in the early 1990s by Edward Oldfield’s group at the NIH [80]. CED involves the infusion of fluids locally under pressure into the interstitial space in the brain or tumor using stereotactically placed catheters. CED primarily utilizes bulk flow, and diffusion is a minor component. While diffusion relies on the concentration gradient, and macromolecules penetrate only up to a few millimeters under diffusive forces, the distribution pattern attained with CED can be described by Darcy’s law, in which the velocity of the molecule is dependent on the pressure gradient and hydraulic conductivity of the medium [81,82].

CED is being widely studied in preclinical and clinical studies for GBM and diffuse intrinsic pontine glioma (DIPG). Souweidane et al. studied CED in combination with PET imaging to deliver a PET-visible histone deacetylase (HDAC) inhibitor, PETobinostat, for theranostic applications in DIPG [83]. Various nanotechnology-based drug delivery systems, like liposomes, nanoparticles, and polymeric micelles, are being administered via CED to increase the volume of the brain tissue accessible to these systems, which are otherwise limited by poor diffusion [84,85,86,87,88,89,90,91]. Models of CED could help inform treatment design and optimization of other parameters like volume of infusate, duration of infusion, catheter design and placement and can guide treatment design [92]. A model was recently developed to understand the flow and distribution of carmustine and paclitaxel solutions and doxorubicin-loaded liposomes post-CED [93,94]. Gill et al. studied CED of panobinostat to understand its pharmacokinetics in brain tissue [95]. A vast number of clinical trials have been initiated to investigate CED for delivery of both large and small molecules, and some of these have been compiled in Table 3. 

CED is a promising technique that has the potential to overcome the limitations posed by systemic delivery. Successful translation of this technique to the clinic would have varied applications to treat a multitude of CNS disorders. For CED to reach its full therapeutic potential, characteristic challenges like catheter design and placement, prevention of reflux, tracking infusate delivery, reduction in mechanical tissue damage and edema, and the potential requirements of multiple infusions need to be addressed. Other challenges associated with CED include cost of the procedure, specific clinical expertise, and postprocedural imaging [86].

**Table 3:** Summary of clinical trials with convection-enhanced delivery (CED) for brain tumors from the clinicaltrials.gov database (accessed on 20 September 2020).

Title	Purpose	NCT Number	Phase	Status/Outcome	Ref
MTX110 by Convection-Enhanced Delivery in Treating Participants With Newly-Diagnosed Diffuse Intrinsic Pontine Glioma (PNOC015)	To study the side effects of panobinostat nanoparticles formulation MTX110 in participants with newly diagnosed DIPG	NCT03566199	I/II	Active, not recruiting	[96]
Chronic Convection Enhanced Delivery of Topotecan	Primarily to establish the safety of prolonged intracerebral CED of chemotherapy in patients with recurrent HGG. Secondly to determine topotecan distribution and radiographic tumor response under the given CED conditions	i. NCT03154996 ii. NCT03927274 iii. NCT02278510 iv. NCT00308165	i. I ii., iii. Early phase I iv. I/II	i.Active, not recruiting ii. Recruiting iii. Completed: Safety of CMC catheters has been reported iv. Recruiting	[97,98,99,100,101]
CED With Irinotecan Liposome Injection Using Real-Time Imaging in Children With Diffuse Intrinsic Pontine Glioma (DIPG; PNOC 009)	Phase I and Early Efficacy Study of CED of irinotecan liposome injection (nal-IRI) using real-time imaging with gadolinium in children with DIPG who have completed focal radiotherapy	NCT03086616	I	Recruiting	[102]
CED of 124I-Omburtamab for Patients With Non-Progressive Diffuse Pontine Gliomas Previously Treated With External Beam Radiation Therapy	To studythe safety of 124I-omburtamab given by CED at different dose levels for DIPG	NCT01502917	I	Recruiting	[103]
CED of MTX110 Newly Diagnosed Diffuse Midline Gliomas	To find the maximum tolerated dose of MTX110 (a water-soluble Panobinostat nanoparticle formulation) and Gadolinium that can be given safely in children with newly DIPG	NCT04264143	I	Recruiting	[104]
Carboplatin in Treating Patients With Recurrent High-Grade Gliomas	To evaluate the safety and toxicity of carboplatin administered by CED in HGG. It is a dose-escalating study.	NCT01644955	I	Completed	[105]
Convection-Enhanced Delivery (CED) of MDNA55 in Adults With Recurrent or Progressive Glioblastoma	Single-arm study with the primary endpoint of median overall survival (mOS) and a secondary endpoint of objective response rate (ORR) following a single intra-tumoral infusion of MDNA55 in adult recurrent GBM subjects	NCT02858895	II	Completed: mOS 12.4 months for all patients vs. 7.2 months in synthetic control arm (SCA); 13.2 months in patients with high IL4R expression vs. 6.1 months in SCA	[106,107,108]
An Open-Label Dose Escalation Safety Study of Convection-Enhanced Delivery of IL13-PE38QQR in Patients With Progressive Pediatric Diffuse Infiltrating Brainstem Glioma and Supratentorial High-Grade Glioma	Test the safety and feasibility of giving IL13-PE38QQR directly into regions of the brain in pediatric patients with DIPG or HGG, using CED	NCT00880061	I	Terminated: did not reach the entire MRI-defined tumor volume in any patient, short-term radiographic effects were observed in 2 of the 5 patients treated.	[109,110]
Study of Convection-Enhanced, Image-Assisted Delivery of Liposomal-Irinotecan In Recurrent High-Grade Glioma	Dose toleration study to determine MTD of nanoliposomal irinotecan in adults with recurrent HGG by CED	NCT02022644	I	Recruiting	[111]
IL13-PE38QQR Infusion After Tumor Resection, Followed by Radiation Therapy With or Without Temozolomide in Patients With Newly Diagnosed Malignant Glioma	Determine the highest dose of IL13-PE38QQR that can be safely administered by CED to the area around the tumor site after surgical resection and concurrent radiation or TMZ	NCT00089427	I	Completed: Positive results, overall survival linked to catheter placement	[112,113]
Safety and Efficacy Study to Treat Recurrent Grade 4 Malignant Brain Tumors	To study the safety and efficacy of TP-38 at 100 ng/mL	NCT00104091	II	Completed: Results pending	[114]
Maximum Tolerated Dose, Safety, and Efficacy of Rhenium Nanoliposomes in Recurrent Glioma (ReSPECT)	A multicenter, sequential cohort, open-label, volume, and dose-escalation study of the safety, tolerability, and distribution of 186RNL given by CED to patients with recurrent or progressive malignant glioma after standard surgical, radiation, and/or chemotherapy treatment	NCT01906385	I/II	Recruiting	[115]
Safety Study of Replication-competent Adenovirus (Delta-24-RGD) in Patients With Recurrent Glioblastoma	To determine the safety and tolerability of Delta-24-RGD administered by CED to the tumor and the surrounding infiltrated brain in patients with recurrent GBM	NCT01582516	I/II	Completed: Safe and robust replication of the AAV, killing of rHGG cells. ≥95% reduction in tumor size in some patients, 5 patients survived >3 years	[116,117]
A Dose-Escalation Phase I Study Of Human-Recombinant Bone Morphogenetic Protein 4 Administered Via CED In GBM Patients	To evaluate the feasibility and safety of intratumor and interstitial therapy with hBMP4 in increasing doses in patients with progressive and/or multiple recurrent GBM	NCT02869243	I	Recruiting	[118]
The PRECISE Trial: Study of IL13-PE38QQR Compared to GLIADEL Wafer in Patients With Recurrent Glioblastoma Multiforme	To determine whether overall survival duration, safety, and quality of life are improved for patients treated with IL13-PE38QQR compared to patients treated with GLIADEL^®^ Wafer following surgical tumor removal in treatment of first recurrence GBM	NCT00076986	III	Completed: There was no survival difference between CB administered via CED and Gliadel^®^ Wafer	[119,120]
Phase 1 Trial of D2C7-IT in Combination With i. 2141-V11 for Recurrent Malignant Glioma ii. Atezolimab for recurrent gliomas	Phase 1 study of D2C7-IT in combination with monoclonal antibodies	i. NCT04547777 ii. NCT04160494 iii. NCT02303678	I	i. Not yet recruiting ii, iii. Recruiting	[121,122,123]
Phase 1b Study PVSRIPO for Recurrent Malignant Glioma in Children	Confirm the safety of the selected dose and potential toxicity of oncolytic poliovirus (PV) immunotherapy with PVSRIPO for pediatric patients with recurrent WHO grade III or IV malignant glioma, to determine MTD for phase 2	i. NCT03043391, ii. NCT01491893, iii. NCT04479241	I	i. Recruiting ii. Active, not recruiting	[124,125,126]
Phase IIb Clinical Trial With TGF-β2 Antisense Compound AP 12009 for Recurrent or Refractory High-Grade Glioma	Multinational dose-finding Phase IIb study of the efficacy and safety of two doses of AP 12009 (OT-101/trabedersen) compared to standard chemotherapy (TMZ or PCV) in adult patients with confirmed recurrent high-grade glioma	NCT00431561	IIb	Completed: OT-101 is an effective agent against recurrent gliomas without the myelosuppression effects of chemotherapy, which is unavailable	[127,128,129]

### 4.3. Biodegradable Wafers

The development of biodegradable polymers for drug delivery has surged ahead in recent decades, and numerous controlled release implants have been developed. Such implants allow precise control over the rate of drug release, prolong local exposure, and reduce systemic toxicities. A major landmark of this technology in brain tumor treatment is the Gliadel^®^ polymeric wafer technology that was approved by the Food and Drug Administration (FDA) in September 1996 as an adjunct to surgery to prolong survival in patients with recurrent GBM, for whom surgical resection is indicated [130,131]. The wafers are surgically implanted at the time of tumor resection and gradually release the loaded DNA and RNA alkylating agent, carmustine (BCNU), with the intention of drug diffusion into surrounding tissue. The polymer is polyanhydride poly[1,3-bis (carboxyphenoxy) propane-co-sebacic-acid] (PCPP:SA), of which more than 70% is biodegraded within three weeks of implantation [131]. Stea et al. conducted a systematic literature review of clinical trials and reports of Gliadel^®^ wafers in combination with radiation therapy and TMZ, and their findings suggest a positive additive effect without an increase in toxicity [132]. This warrants the need for larger prospective trials that combine Gliadel^®^ with TMZ and radiation therapy with scientifically backed study design and patient selection for confirming and establishing the anticipated synergy between these treatments [133,134,135,136,137,138,139,140].

Recent research efforts are examining the efficacy of wafers after coloading BCNU and TMZ to determine if there is prolonged survival compared to wafers of the individually loaded drug or orally administered TMZ in rodent glioma models. These PLGA wafers used a pre-encapsulation process and reported 25% long-term survivors (survived >120 days compared to median survival of 28 days) in the F344 rat model [141]. Other chemotherapeutic agents that have been explored for sustained local delivery via polymeric implants include taxol, camptothecin, minocycline, doxorubicin, and others [142,143,144,145,146,147,148,149,150,151,152,153,154,155,156]. Novel polymeric implants and microchips can be used to deliver several drugs locally at varying time points in a controlled manner, and various formulations such as nanoparticles, liposomes, and microparticles can also be delivered via these implants.

A major challenge for this technology is to ensure biocompatibility and biodegradation, as there are reports of incompletely biodegraded materials found up to 68 weeks after implantation; hence, patients must be monitored carefully [157]. The success of these therapies is limited due to their inability to reach distant, invasive, and dense tumor cells due to poor diffusion characteristics. Focused treatments, in combination with localized delivery, are required to target these cells, and local delivery via implants will play a critical role in this mode of drug delivery [158,159].

## 5. Blood–Brain Barrier Disrupting Strategies

Apart from these invasive methods, other noninvasive techniques have been investigated to transiently disrupt the neurovasculature to enhance drug delivery to the CNS. These methods may have better patient compatibility compared to invasive approaches such as CED and IT injection and will perhaps allow lower dosage, thus reducing toxicity compared to traditional systemic administration routes like intravenous injection.

### 5.1. Osmotic Blood-Brain Barrier Disruption

**Figure 4:**
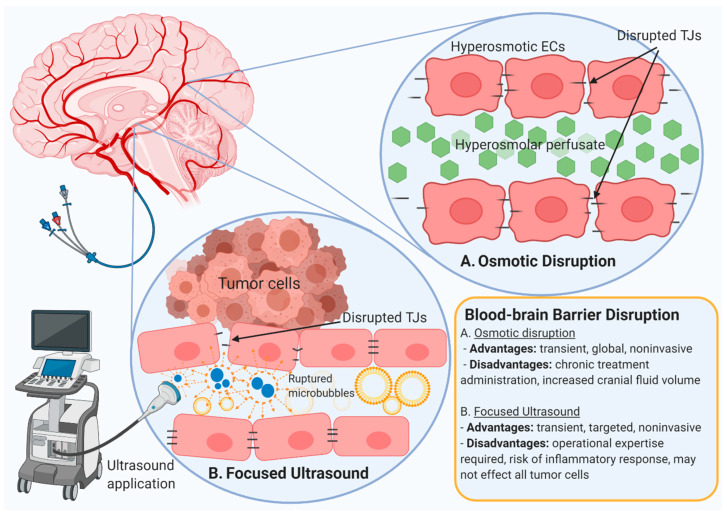
Drug delivery to the brain may be increased by noninvasive BBB disruption (BBBD) techniques, including (**A**) osmotic disruption and (**B**) focused ultrasound. In osmotic disruption, infusion of a hyperosmolar solution via a cerebral artery results in endothelial cell shrinkage, temporarily disrupting tight junctions. Focused ultrasound uses an infusion of inert gas-filled microbubbles, which, upon application of focused ultrasound, may burst and temporarily disrupt tight junction proteins. Advantages and disadvantages of both are listed.

The tight junctions of the cerebrovascular endothelium can be transiently and reversibly disrupted by the infusion of a hyperosmolar solution into a cerebral artery putatively because of the shrinkage of endothelial cells following the splitting of tight junctions. The resulting intracellular spaces increase paracellular diffusion and facilitate the delivery of therapeutic, diagnostic, and functional agents relevant to CNS disease. This method was first proposed by Rapoport et al. in 1972, who exposed the pia arachnoid surface of the cerebral cortex of healthy rabbits to different osmotic concentrations, resulting in osmotically induced and reversible cell shrinkage (Figure 4) [160]. In practice, this method involves the infusion of 1.4 M mannitol, which has been FDA-approved for administration to patients [161]. Besides mannitol, other hypertonic solutions used for transient barrier disruption include arabinose, lactamide, saline, urea, and several radiographic contrast agents [162]. The first Phase I clinical trial on osmotic BBB disruption (BBBD) for enhanced drug delivery to the brain was initiated in 1979 [163]. Using this technique in experimental and clinical treatment of brain tumors, permeability enhancements of greater magnitude were observed for tumors with low, rather than high, initial permeability relative to that of normal brain [164].

In the clinical setting, chemotherapeutics used in combination with osmotic BBBD include methotrexate (MTX), carboplatin, melphalan, cyclophosphamide, etoposide, and etoposide phosphate [165]. A clinical study by Neuwelt et al. during the 1980s demonstrated that osmotic BBBD plus MTX produced long-term remission and improved survival in patients with primary CNS lymphoma (PCNSL) [166]. Another clinical study from 1982 to 2005, involving more PCNSL patients treated with osmotic BBBD and methotrexate at four institutions, showed durable tumor control over a 23-year period [167]. Additional clinical studies have demonstrated relatively low toxicity [168,169].

Although the disruption is transient and is fully reversed within several hours [170,171], one risk of osmotic BBB disruption is the additional mass effect in the brain that results from a 1.5% increase in brain fluid content [172,173]. Assessment of the extent of the tumor and the associated mass effect prior to osmotic BBB disruption is important for optimizing protocols and minimizing the risks of this procedure [174].

### 5.2. Microbubble-Mediated Focused Ultrasound

A localized disruption of the neurovasculature using focused ultrasound (FUS) has been suggested as an anatomically or functionally targeted method for drug delivery from the vasculature into the brain parenchyma. FUS-induced BBB opening in the presence of microbubbles is local, transient, and reversible, usually within several hours [175]. Its feasibility and efficacy to promote the delivery of therapeutic agents into the brain have been examined extensively since 1997 when Kullervo Hynynen and Ferenc Jolesz first demonstrated the potential feasibility of BBBD through the intact human skull utilizing short, high-intensity ultrasound [176]. More recently, with the application of magnetic resonance-compatible transducers, image-guided FUS has allowed targeted localization to brain tumors and reduced the risk of off-target effects [177,178,179].

Therapeutic FUS is generally applied in conjunction with intravenously administered microbubbles (Figure 4). These microbubbles are lipid, protein, or polymer-shelled, inert gas-filled bubbles that are usually between 0.5 to 10 µm in diameter [180]. They are currently FDA-approved for use as contrast agents in ultrasound imaging and are utilized in the context of drug delivery to help reduce the energy threshold required for BBBD [181]. The energy threshold is, to some extent, determined by the size of microbubbles, and, typically, the smaller the diameter of these microbubbles, the higher the pressure required for effectiveness [182]. It is important to carefully control the energy level of FUS, as high pressure and frequency may cause an inflammatory response and/or tissue damage, such as hemorrhage and apoptotic neuronal damage [183]. Extensive research into the safety and feasibility of FUS has been initiated in a variety of CNS diseases, and recently, clinical studies have been conducted to determine the safety and efficacy of the application of FUS with intravenously injected microbubbles in human brain tumors [184,185,186].

FUS with microbubbles has been applied to glioma treatment to assist in the delivery of free therapeutic agents, including small molecules such as doxorubicin [187,188], as well as large molecules like bevacizumab and trastuzumab [189,190,191]. It has also been used for gene- [192,193] and drug-loaded nanoparticle delivery [192,193,194,195]. An alternative way to increase the penetration of therapeutic drugs into the brain is to load the drug of interest into the microbubbles themselves [196,197]. These studies demonstrated that the drug release process could be controlled by the acoustic emission provided by ultrasound imaging. FUS has also been combined with magnetic targeting of magnetic nanoparticles (MNPs) in conjunction with MRI monitoring for CNS drug delivery [198,199]. In this synergistic system, FUS facilitates delivery through the vascular wall via passive enhanced permeability and retention (EPR) effects, while magnetic forces actively enhance the deposition of MNPs into the brain.

This approach for BBBD has been shown to be relatively safe, but it also has limitations [200]. Despite the early increase in drug delivery to the CNS, recent studies with large molecules showed that enhanced permeability was diminished after 5 days [201]. This could be one explanation for why many animal studies on FUS conducted with trastuzumab showed nonsignificant differences regarding survival when comparing FUS- and non-FUS-treated groups [189,190]. Repeated FUS treatment before drug administration may, therefore, be required, which increases risk. Other obstacles in a wide clinical FUS application include issues with repeatability of the FUS procedure and dependence on MRI and specially-trained operators [202].

Overall, FUS-mediated BBBD has provided a promising approach to therapeutic delivery to brain tumors and other CNS diseases such as Alzheimer’s and Parkinson’s diseases [203]. Meanwhile, successful and wider clinical translation requires a more extensive and thorough examination of possible safety issues due to repeated BBBD, the repeatability of FUS treatment, and optimization of ultrasound parameter settings.

## 6. Nanoparticles

Nanoparticles are a large category of nanoscale particles (1–1000 nm) with the capacity to adsorb, entrap, or be modified with various therapeutic agents. These particles are promising strategies to improve brain drug delivery [204]. This section is focused on nanoparticle strategies to overcome low neurovascular permeability and increase drug delivery into brain tumors. A summary of the major categories of the current nanoparticles is shown in Table 4 and can be broadly categorized into biological vectors and synthetic vectors of various types.

### 6.1. Biological Vectors

#### 6.1.1. Viral Vectors

Viral vectors have been repeatedly used in GBM gene therapy clinical trials [205]. Viral vectors have the ability to naturally infect cells with nucleic acids with high transfection efficiency [206]. Currently, several viruses have been developed into vectors for brain delivery, including retroviruses, adenoviruses, and adeno-associated viruses (AAVs) [13,205,207,208,209]. Although viral vectors have been studied for over two decades, they have only resulted in marginal increases in overall survival. Long et al. undertook a Phase I clinical trial of p53 gene therapy using an adenovirus vector (Ad-p53). However, transfected cells were found residing only around the injection site [209]. The limitations of using viral vectors for drug delivery include poor brain tumor penetration, highly invasive administration methods, and a prevailing risk of oncogenesis and lethality of viral vectors [13,205,207,208,209].

#### 6.1.2. Exosomes

Exosomes are small endogenous extracellular vesicles (40–100 nm in diameter) that are secreted by various types of cells and have drug-loading and signal-carrying capacity [210]. Exosomes can be loaded with various kinds of cargos, such as nucleic acids, proteins, and small molecules, due to their bubble-like structure [211,212]. Exosomes are generally stable in circulation and lack significant immunogenicity [13,212]. They transport cargos among cells and may even cross BBB via endogenous pathways of intercellular communication [13,210,211,212]. In addition, exosomes have also played important roles in cancer immunotherapy by virtue of the biological signals enclosed in exosomes [210,213]. However, the technologies and strategies to isolate and purify exosomes must be further developed to ensure quality control, and other side effects such as the potential tumor induction risk of tumor-cell-derived exosomes have to be taken into account [206,210,212].

#### 6.1.3. Cell Delivery

Cell-based drug delivery is another exciting strategy for the delivery of therapeutics across the BBB via the innate mobility of cells. There are two cell types that have been evaluated as therapeutic carriers: immune cells and stem cells. In particular, neural stem cells (NSCs), mesenchymal stem cells (MSCs), and neutrophils have been studied for cell-based therapy [214,215,216]. These cell carriers can deliver a variety of therapeutics, including genes, cytokines, enzymes, and nanoparticles across the BBB and are naturally recruited to the sites of brain tumors by an inflammation-mediated pathway [217]. Xue et al. demonstrated that neutrophil-mediated paclitaxel cationic liposomes could penetrate the brain efficiently and slowed the recurrent growth of tumors in mice, with significantly improved survival rates [216]. Balyasnikova et al. developed engineered neural stem cells to express membrane-bound TNF-α-related apoptosis-inducing ligands to induce apoptosis in glioma cells [218]. Detailed mechanisms of the cell carrier’s delivery can be found in the recommended reviews [217,219]. The major difficulties associated with this strategy are the limited therapeutics loading and potential toxicity of the cargo to the cell carriers themselves. Moreover, the spatial and temporal release of the therapeutic agents from the cell carriers must be well-controlled during drug delivery in order to achieve the expected efficacy [217,220].

### 6.2. Synthetic Vehicles

Synthetic nanoparticles have been broadly investigated to deliver drugs to the brain. The physicochemical properties of the nanoparticles, including size, surface charge, and lipophilicity, are important in the brain passive-diffusion process. A growing interest in the application of inorganic nanoparticles, especially metallic nanoparticles and metallic oxide nanoparticles, in CNS delivery has emerged among the BBB research community [221,222]. Iron oxide nanoparticles, such as maghemite (γ-Fe_2_O_3_) and magnetite (Fe_3_O_4_), are extensively explored due to their inherent magnetic properties, coupled with tunable size and surface functionality [223,224,225]. Mesoporous silica nanoparticles (MSNPs) are nanoscale silica particles with good loading capacity due to their porous structure and easily modified surface; they are the most commonly applied silica-based delivery vehicles [226]. These inorganic nanoparticles are produced on the scale of nanometers in order to increase their ability to cross the BBB, providing photodynamic or contrast imaging functions due to material properties [223]. However, the potential for neurotoxicity and unspecific distribution are serious barriers to the broad application of metallic nanoparticles [224].

Actively targeted nanoparticles account for the majority of brain drug delivery systems currently under investigation. Surface-modified nanoparticles are transported into the brain, bypassing the BBB by three main routes: adsorptive-mediated transcytosis (AMT), receptor-mediated transcytosis (RMT), and transporter-mediated transcytosis (TMT) [219,227]. Adsorptive-mediated brain targeting largely depends on the electrostatic interaction between the positively-charged drug delivery systems and the negatively-charged BBB [228]. In 2007, Lu et al. evaluated the transcytosis of cationic bovine serum albumin conjugated poly(ethyleneglycol)-poly(lactide) (PEG-PLA) nanoparticles (CBSA-NP) on the BBB. They found that the transcytosis ability of CBSA-NP increased with the increase of CBSA surface density per nanoparticle [229]. However, this nonspecific targeting is the inherent limitation of AMT since negatively charged membranes are present throughout all the vascular system [219]. Moreover, positive nanoparticles have more tendency to adsorb surrounding proteins and form protein coronas [230]. RMT and TMT target the brain more specifically than AMT through ligand-receptor recognition. These receptor-mediated strategies will be further introduced in the following section. The transporters for TMT are usually transporters of nutrient materials like sugars, vitamins, hormones, and amino acids [219,231,232]. These actively targeted nanoparticles target brain tumors more specifically and, therefore, have higher accumulation and lower systemic side effects. However, some concerns such as protein adsorption and corona formation around the nanoparticles, potential neurotoxicity, and difficulty of manufacturing due to the complex structures need to be further addressed [219,233].

**Table 4:** Nanoparticles for brain drug delivery.

Ref	[207,208,209]	[210,211,213]	[214,215,216,218]	[221,222]	[227,228]	[230,233,234] [231,235]
Examples	AAV9-hIFNβ, retroviral herpes simplex virus-thymidine kinase (HSV-tk), Toca 511 delivers suicide gene, cytosine deaminase (CD), and in combination with oral prodrug, adenoviral vector carrying the wild-type p53 gene (Ad-p53)	Paclitaxel with bEND.3 cell-derived exosome, doxorubicin with U-87 MG cell-derived exosome, miRNA-486-5p transferred exosomes, siKrasG12D iExosomes, tumor-cell-derived exosomes and α-GalCer on a DC-based vaccine	Neutrophil-mediated paclitaxel cationic liposomes, carboxylesterase-expressing allogeneic neural stem cells, bone morphogenetic protein 4 (BMP4) expressing adipose-derived mesenchymal stem cells, neural stem cells engineered to express membrane-bound TRAIL (NSCs-mTRAIL)	Iron oxide nanoparticles, gold nanoclusters, mesoporous silica nanoparticles, lanthanide upconversion particles	Cationized bovine serum albumin modified NPs, polysorbate 80 or poloxamer 188 overcoated NPs, apolipoprotein bound nanoparticles,	Transferrin receptor-targeted (OX26) immunoliposomes, LDLR-DHA nanoparticles, insulin-mAb-modified HSA NPs; Glutathione-modified liposomes, choline-derivate-modified NPs
Disadvantages	i. Limited brain tumor penetration ii. Highly invasive administration method iii. Prevailing risk of oncogenesis and lethality	i. Lacking standardized isolation and purification procedure, ii. Donor cells choice iii. Potential tumor induction risk of tumor cell-derived exosomes	i. Potentially toxic effects of the cargo on the cell carrier itself ii. Spatial and temporal release of the therapeutic agent iii. Limited loading efficiency	i. Neurotoxicity ii. Unspecific distribution	i. Poor selectivity ii. Protein adsorption and corona formation	i. Protein adsorption and corona formation ii. Potential neurotoxicity iii. Difficulty of manufacturing
Advantages	i. High efficiency for gene delivery, ii. Innate ability to infect cells	i. Nonimmunogenic ii. Stable and long circulation iii. Cross BBB iv. Target the tissue via their natural surface proteins	i. Cross BBB ii. Naturally recruited to sites of brain tumors	i. Ultrasmall size ii. Easily modified iii. Contrast imaging iv. Phototherapeutics	i. Electrostatic adsorption ii. Improve cellular uptake iii. Improve penetrating efficiency	i. High selectivity ii. Enhanced brain accumulation iii. Cross BBB iv. Decrease systemic toxicity
Strategy					AMT	RMT and TMT
	Viral vectors	Exosomes	Cell carriers	Passive diffusion	Actively targeted delivery	
	Biological vectors			Synthetic vehicles		

## 7. Receptor-Mediated Transcytosis

**Figure 5:**
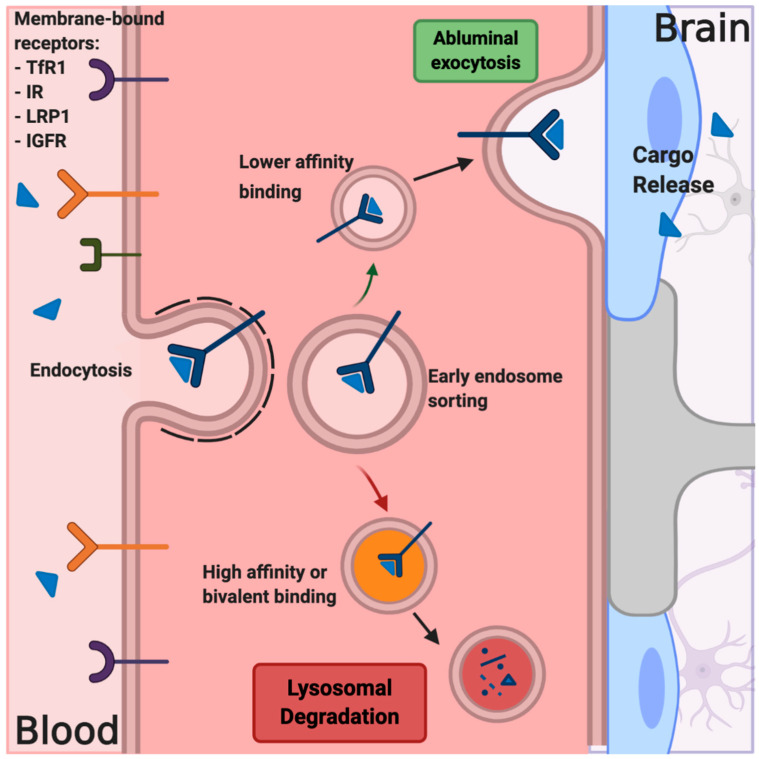
Receptor-mediated transcytosis is one of the most common techniques to increase the delivery of large molecules, nanoparticles, and brain-impermeant drugs to brain tumors. Cargo bound to endothelial-membrane-bound receptors is pulled into endothelial cells (ECs) and sorted in the early endosome. Bivalent-binding and high-affinity cargo-receptor complexes are often trafficked to the lysosome for degradation, whereas cargoes bound with lower affinity are more likely to be trafficked for transport across the cell. The cargo is then released on the abluminal side of the endothelium, and the receptor may be recycled back to the luminal membrane.

The final strategy to be discussed in this section is the use of endogenous active transport mechanisms to improve drug delivery to brain tumors, in particular, RMT. This transport is accomplished by three basic steps: binding of the cargo to the target receptor on the luminal side of the brain EC; endocytosis, sorting, and transport across the EC cytoplasm; release of the cargo from the basolateral membrane of the EC into the brain interstitium (Figure 5). As discussed in reference to the heterogeneity of BTB permeability, it may be beneficial to address the treatment of brain tumors by utilizing a “whole brain” delivery strategy to address intact NVU/BBB [236]. RMT-based strategies can be viewed as such an approach because they are generally designed to target transport mechanisms that are functional throughout the extensive vasculature of the brain. Successful brain delivery using RMT requires that target receptors have a high relative expression on the luminal side of the brain endothelium, must mediate transcytosis, and should have high turnover [237,238]. Importantly, the receptor-binding moiety should also have a relatively low affinity for the target in order to optimize drug delivery and release in the brain parenchyma and to limit trafficking to the lysosome [237,239].

Common receptor targets at the BBB include transferrin receptors (TfR1), insulin receptors (IRs), insulin-like growth factor receptors (IGFRs), the low-density lipoprotein-related protein receptor 1 (LRP1), and nicotinic acetylcholine receptors (nAchRs) [240,241]. This strategy has been widely explored in the brain barriers research community for use in a number of CNS diseases, from brain tumors to lysosomal storage disorders, Alzheimer’s disease, Parkinson’s disease, and others. As noted in the previous sections, these various RMT-based delivery mechanisms are often combined with other technologies like BBBD, CED, nanoparticle formulations, gene delivery, and novel biologics. Herein, we will classify delivery constructs into two categories: shuttle peptides and antibody-based constructs.

### 7.1. BBB Shuttle Peptides

Shuttle peptides are relatively short sequences of amino acids (<50 AAs) that bind to a receptor on the luminal side of the EC to induce endocytosis of the cargo. They are often based on the known sequences from receptor-binding domains of endogenously transported substances like insulin, ApoE, and transferrin, but they may also be discovered by phage display biopanning. These peptides can be directly bound to cargo or associated via noncovalent interactions [238]. Covalently bound shuttle peptides are more likely to have known and relatively consistent stoichiometry, kinetics, and affinity. On the other hand, some investigation into noncovalent associations that may be more prone to cargo release might be more rapidly translated across a number of different drugs for various applications [242,243]. However, their binding affinity for cargo and optimal stoichiometry must be determined. The benefits of shuttle peptides, in general, include their relatively small size, simplicity of synthesis and purification, versatility, and discovery through biopanning. Their limitations include their liability to proteolytic degradation and relatively short half-life in circulation, which may be ameliorated somewhat by cyclization [244].

All of the aforementioned receptors have been targeted for drug delivery. Though there is debate as to the location of LRP1 expression on brain ECs [245,246], a number of shuttle peptides have been developed to target this receptor, including agiopeps and K16ApoE. The K16ApoE peptide consists of 16 lysine residues and the LRP1-binding domain of ApoE [243,247]. While this has shown some evidence of improved drug delivery to the brain, the therapeutic window is narrow and may not be suitable for clinical translation due to acute toxicities observed in mice [242]. Angiopep-2 is one of the most well-characterized shuttle peptides for brain delivery, and it is derived from the Kunitz domain of aprotinin [248]. Angiopep-2 has been widely utilized as a targeting moiety for nanoparticle formulations of antineoplastic agents like TMZ and docetaxel, as well as siRNA, monoclonal antibodies (mAbs), and various radiosensitizing agents for the treatment of CNS tumors [249,250,251,252,253,254,255]. The most developed shuttle peptide construct is likely ANG1005, an angiopep–paclitaxel conjugate recently investigated in clinical trials for the treatment of brain metastases from breast cancer, as well as meningiomas [256,257,258]. Other shuttle peptides include peptide-22, which binds to LDLR, and glutathione, which binds to the GSH transporter. GSH-coated pegylated nanoparticles show increased CNS penetration and have been investigated in clinical trials for the delivery of doxorubicin to brain tumors [259,260]. TfR1 has also been widely investigated as a delivery mechanism due to its expression in tumor cells and brain ECs [261]. T7 targets TfR1 and is a shuttle peptide that has been investigated to deliver antisense oligonucleotides to gliomas [262,263]. Delivery of radiosensitizing gold nanoparticles to brain tumors has been shown to be enhanced by Tfpep [264], and another TfR1-directed peptide, THR, was recently compared with other previously mentioned peptides for the delivery of AAVs and gold nanoparticles to the brain but without specific applications towards the treatment of brain tumors [224,265]. A vast variety of BBB shuttle peptides have been explored, and we direct the reader to an excellent review of the topic for further reading [238].

### 7.2. Antibody-Based Delivery Systems

Antibody-based therapies are one of the most rapidly evolving fields in pharmaceutics due to their plasma stability, long half-life, and specificity. Antibodies, specifically immunoglobulin G (IgG), are large, bivalent molecules (~150kDa) composed of two identical heavy chains and two identical light chains bound by disulfide bonds. These proteins have proved to be incredibly effective in the treatment of peripheral tumors, but they do not generally cross the BBB from blood into brain [265,266,267,268]. In fact, without enhanced delivery mechanisms, drug accumulation in the brain is likely to be much less than 1% [266,269]. However, there is still significant interest in delivering these drugs to the brain [24,245,270,271,272], and antibodies are also well-suited to serve the same purpose as shuttle peptides to promote RMT. With the recent blossoming of innovative protein engineering and the use of antibody fragments, the somewhat “modular” structure of IgGs has been exploited to modify and utilize different domains. This allows them to be optimized for use as brain-targeted therapies and brain delivery vehicles.

A variety of therapeutic antibodies and antibody-decorated NPs have been targeted at the brain via the TfRs and IRs for treatments of CNS diseases, most notably Alzheimer’s disease and brain tumors [273,274]. In recent years, a nanocarrier of p53 gene therapy, decorated with anti-TfR1 single-chain variable fragments (scFvs), SGT-53, has been successful in preclinical studies and has moved into clinical trials [275,276,277]. Although a study in adult refractory CNS tumors was terminated, actively recruiting studies for children with refractory solid tumors and planned clinical trials in refractory CNS tumors in pediatric patients (NCT02354547, NCT03554707) are still ongoing. Other imaginative antibody constructs explore bispecific or multivalent targeting [278,279]. Recent work from AbbVie demonstrates the targeting of multivalent, dual-variable-domain IgGs (DVD-Igs) with a dual affinity for precision targeting. These molecules can bind two targets, TfR1 for RMT and HER2, for prospective targeting to HER2+ brain tumors while maintaining the Fc domain unchanged, allowing for beneficial FcRn recycling [280]. Furthermore, recently published work from Denali Therapeutics demonstrates a novel protein transport vehicle (TV) with an affinity for TfR1 incorporated into the Fc region of the IgG, allowing for retention of bivalent binding to the therapeutic target [281,282]. Although these are not explicitly intended to treat brain tumors, they are an exciting contribution.

## 8. Conclusions and Future Perspectives

In the past decade, there has been a tremendous increase in the understanding of the physiology of the BBB. However, this has not translated to efficacious treatment of CNS disorders, which range from epilepsy to brain tumors. In the case of both primary and metastatic brain tumors, the BBB is disrupted heterogeneously, leading to the formation of the blood–tumor barrier (BTB). The BTB harbors considerable structural and functional heterogeneity within the tumor microenvironment and varies across different cancer subtypes [17]. It compels us to question if the leakiness can be used to our advantage to deliver drugs in desired concentrations to the target site.

While some reports have shown a positive correlation between increased permeability in the tumor to tumor size and growth patterns, there are reports, including those from NIH, that demonstrate no correlation between the two. These inconsistencies highlight the problem of heterogeneity of the BBB breakdown, and this challenge is encountered in the clinical setting as well. Diagnosis of brain tumors using fluorescent tracers is facilitated by the increased permeability of the BTB, but why does it not extrapolate to the treatment modalities like chemotherapy? Instead of relying on the altered BBB permeability to deliver cytotoxic cargo to the tumor cells, it would be better to prepare the delivery systems to face the most challenging barrier—the intact BBB—and strategize the delivery to efficiently target the tumor cells and reduce any off-target toxicity.

Over years of research, various strategies have been developed to invasively or noninvasively overcome the BBB. The invasive strategies bypass the BBB altogether and deliver the therapeutic agents directly into the brain parenchyma or into the CSF. These strategies prevent systemic exposure of the drug, thereby limiting its toxicity and side-effects. They have been widely explored in the clinical setting and there are numerous on-going clinical trials, demonstrating the huge potential of this strategy. However, invasive procedures need highly specialized instruments and personnel. From a patient’s perspective, noninvasive strategies are preferred. Various noninvasive BBB-disrupting strategies and nanoparticle drug delivery systems that bypass the BBB by a number of transport routes are discussed in the review. Transient disruption of the BBB using focused ultrasound enables the delivery of a wide range of therapeutics to the brain, ranging from small molecules to large molecules. It is imperative to understand the kinetics and time duration of the temporary disruption to effectively plan the delivery of therapeutics. The first decade of the 21st century saw the “nanoparticle boom” and nanoparticles proved to be able to deliver conventional drugs, recombinant proteins, vaccines, and nucleotides. This versatile carrier system can be modified to target various transcytosis pathways to ensure improved drug delivery using the enhanced permeation retention (EPR) effect [283].

Understanding the physiology of the BBB at the cellular and molecular levels helps researchers design delivery systems that selectively target the receptors and transporters on the cell surface of the BBB. A challenge while developing these constructs is to avoid off-target effects. Ensuring delivery at the site of action is critical to achieving the desired concentrations at that site and minimal off-target effects. Thus, understanding the pharmacokinetics of the delivery systems would be imperative for their progress from the preclinical research settings to the clinical scenario.

In conclusion, a better understanding of the BBB/BTB physiology has led to the development of a multitude of strategies to target the tumor cells present beyond these barriers. As has been demonstrated on numerous occasions in the past, a “one size fits all” approach is not effective. A rational combination of drugs and their delivery is now being designed to attain optimal concentrations in brain tumors. In this way, a comprehensive treatment regime will be established.

## Figures and Tables

**Table 1:** Endothelial cell membrane transporters: partial list of common carriers.

Transport System	Typical Substrate	SLC Family	Common Name
Carbohydrates			
Hexose	Glucose	SLC2A1	Glut1
Sodium Myo-inositol	Myo-inositol	SLC5A3	SMIT
Monocarboxylates			
Monocarboxylic acid	Lactic acid ketones	SLC16A1	MCT1
Amino Acids			
Large neutral amino acid	Phenylalanine	SLC7A5	LAT1
Small neutral amino acid	Alanine	SLC38A2	SNAT2, -3, -5
Cationic amino acid	Lysine	SLC7A1	Cat1, CAT3
Beta amino acid	Taurine	SLC6A6	TauT
Ala-Ser-Cys	Ala, ser, cys	SLC1A4	ASCT1, -2
Excitatory amino acid	Glutamic acid	SLC1A2	EAAT-1, -2, -3
Glycine	Glycine	SLC6A9, A5	GT-1
Others			
Fatty acids	Essential FA LPC-PC (DHA)	SLC44A1/2 Mfsd2A	FATP-1, -4 Mfsd2A
Nucleoside	Adenosine	SLC29A1 SLC28A1	ENT-1, -2; CNT1–3
Hormones	Thyroid T3 Thyroid T4	SLC16A2 OATP1C1	MCT8 OATP1C1
Biotin, pantothenic acid	biotin	SLC5A6	SMVT
Folic acid	Folinic acid	SLC46A1	PCFT
Copper	Cu^+^	SLC31A1	CTR1

**Table 2:** Brain endothelial cell transporters of xenobiotics/drugs. Members of the ABC (ATP-binding cassette) superfamily of transporters demonstrated in brain endothelial cells and non-ABC transporters of organic chemicals potentially present are listed.

Transport System	Common Name	Typical Substrate
ATP Binding Cassette Transporter (ABC)		
ABCB1	P-gp	Broad-spectrum, xenobiotics
ABCG2	BCRP	mitoxantrone anthracycline xenobiotics
ABCC1	MRP1	GSSG, leukotrienes
ABCC5	MRP5	Thiopurines, cyclic nucleotides
ABCC4	MRP4	Organic anions
Non-ABC Transporters		
SLC22A7	OAT2-3	Organic ions
SLC22A8	OATP1A4	
SLC20A2	OATP2B1	
SLCO1A4	OCTN2	
SLCO2B1	OCT1-3	

## Abbreviations

AAV	Adeno-associated virus
ABC	ATP-binding cassette protein
AMT	Adsorptive-mediated transcytosis
BBB	Blood–brain barrier
BBBD	Blood–brain barrier disruption
BCRP	Breast cancer resistance protein
BTB	Blood–tumor barrier
CED	Convection-enhanced delivery
CNS	Central nervous system
CSF	Cerebrospinal fluid
CVO	Circumventricular organs
DIPG	Diffuse intrinsic pontine glioma
EC	Endothelial cells
EGFR	Epidermal growth factor receptor
FDA	Food and Drug Administration
FUS	Focused ultrasound
GBM	Glioblastoma miltiforme
GLUT1	Glucose transporter 1
HDAC	Histone deacetylase
IGFR	Insulin-like growth factor receptor
IR	Insulin receptor
IT	Intrathecal
LDLR	Low-density lipoprotein receptor
LRP1	Low-density lipoprotein-related protein 1
MCT1	Monocarboxylate transporter 1
MNP	Magnetic nanoparticles
MSC	Mesenchymal stem cells
MRI	Magnetic resonance imaging
MRP	Multidrug resistance protein
NSC	Neural stem cell
NVU	Neurovascular unit
NVU/BBB	Neurovascular unit/blood–brain barrier
PDGFR-β	Platelet-derived growth factor-β
P-gp	P-glycoprotein
PLGA	poly(lactic-co-glycolic acid)
RMT	Receptor-mediated transcytosis
TfR1	Transferrin receptor 1
TMT	Transporter-mediated transcytosis
VEGF	Vascular endothelial growth factor
